# Identification of trunk mutations in gastric carcinoma: a case study

**DOI:** 10.1186/s12920-017-0285-y

**Published:** 2017-07-17

**Authors:** Zhan Zhou, Shanshan Wu, Jun Lai, Yuan Shi, Chixiao Qiu, Zhe Chen, Yufeng Wang, Xun Gu, Jie Zhou, Shuqing Chen

**Affiliations:** 1 College of Pharmaceutical Sciences, Zhejiang University, Zhejiang, Hangzhou 310058 China; 2 Zhejiang Hospital of Traditional Chinese Medicine, Zhejiang, Hangzhou 310058 China; 3 Department of Biology and South Texas Center for Emerging Infectious Diseases, University of Texas at San Antonio, San Antonio, TX 78249 USA; 4 Department of Genetics, Development and Cell Biology, Iowa State University, Ames, IA 50011 USA; 5 International Center for Precision Medicine, Zhejiang California International NanoSystems Institute, Zhejiang, Hangzhou 310058 China

**Keywords:** Intratumor heterogeneity, Gastric carcinoma, Multiregional sampling, Cancer somatic mutation, Cancer evolution

## Abstract

**Background:**

Intratumor heterogeneity (ITH) poses an urgent challenge for cancer precision medicine because it can cause drug resistance against cancer target therapy and immunotherapy. The search for trunk mutations that are present in all cancer cells is therefore critical for each patient.

**Case presentation:**

In this study, we aimed to evaluate the efficiency of multiregional sequencing for the identification of trunk mutations present in all regions of a tumor as a case study. We applied multiregional whole-exome sequencing (WES) to investigate the genetic heterogeneity and homogeneity of a case of gastric carcinoma. Approximately 83% of common missense mutations present in two samples and approximately 89% of common missense mutations present in three samples were trunk mutations. Notably, trunk mutations appeared to have higher variant allele frequencies (VAFs) than non-trunk mutations.

**Conclusions:**

Our results indicate that small-scale multiregional sampling and subsequent screening of low VAF somatic mutations might be a cost-effective strategy for identifying the majority of trunk mutations in gastric carcinoma.

**Electronic supplementary material:**

The online version of this article (doi:10.1186/s12920-017-0285-y) contains supplementary material, which is available to authorized users.

## Background

Cancer is believed to be driven by somatic mutations, including single nucleotide variations (SNVs), small insertions/deletions (INDELs), copy number variations, structural variations, and epigenetic changes [1, 2]. In addition to their role in oncogenesis, cancer somatic mutations are potential biomarkers for cancer diagnosis and target therapy [3, 4]. As long as the somatic mutation results in the production of new epitopes on the membrane of the tumor cell, the active protein arising from the mutation will cause the immune system to recognize the affected cell as foreign. Thus, such proteins are considered ideal targets for cancer immunotherapy, which is now called neoantigen [5]. With the development of cancer genomics, tumor-specific neoantigens have attracted much attention in current biomedical research because of their potential to be ideal targets for cancer immunotherapy [6–10]. Unfortunately, large-scale cancer genome sequencing analyses have revealed that cancer is a heterogeneous disease and that no two cancers harbor the same complement of somatic mutations. Additionally, there is no common set of mutated genes in all cancers [11–13]. Intertumor heterogeneity suggests that cancer is a personalized disease. Only a very small number of genes (such as TP53) are recurrently mutated in more than 10% of cancer patients [14]. Therefore, theoretically, somatic mutations should be analyzed for each individual cancer patient to conduct precision diagnosis and precision treatment.

Furthermore, recent studies involving multiregional genome sequencing and single-cell sequencing in different tumor types have revealed that cancer is not only heterogeneous between tumors but also highly heterogeneous within tumors [15–21]. Intratumor heterogeneity (ITH) is a major challenge to cancer precision medicine because it might lead to an underestimation of the cancer somatic mutation landscape based on a single tumor biopsy and might contribute to failure in drug treatment and the emergence of drug resistance [22]. Therefore, a single tumor biopsy might be insufficient for identifying all cancer somatic mutations in that tumor because such a biopsy cannot distinguish trunk mutations that are present in all regions of the tumor, branch mutations that are present in only some regions of the tumor, and private branch mutations that are present only in one region of the tumor [15]. Cancer is widely accepted to be a microevolutionary process that originates from a single cell [2, 23]. Determining the phylogeny of cancer evolution will help identify trunk, branch, and private branch mutations. The identification of trunk mutations is critical for biomarker development and cancer precision medicine, because these mutations represent the genomic differences between all cancer cells and normal cells. Specifically, trunk mutations within protein-coding regions might result in mutant proteins, which are potential tumor-specific neoantigens in all cancer cells.

In this study, multiregional whole-exome sequencing (WES) was applied to identify the intratumor heterogeneity and homogeneity of gastric carcinoma through a case study. We classified these mutations into trunk, branch, and private branch mutations by comparing the somatic nonsynonymous substitutions within six different regions of the primary tumor from a male gastric cancer patient. Private branch mutations show ITH, whereas trunk mutations indicate homogeneity. We further discussed the most cost-effective number of regions required to identify the majority of trunk mutations and compared the mutation frequencies between trunk and non-trunk mutations based on both our case study and published data for multiregional samples of 11 surgically resected lung adenocarcinomas [15].

## Case presentation

**Fig. 1:**
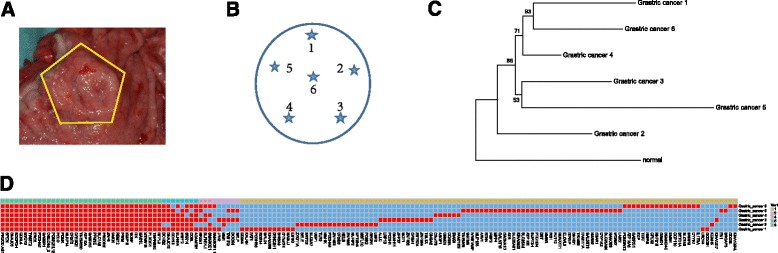
Genetic intratumor heterogeneity, homogeneity, and phylogeny in a patient with gastric carcinoma. **a**-**b** The biopsy sites and regions harvested clockwise from the surgically resected gastric carcinoma. **c** Phylogenetic relationships among six tumor regions. **d** Distribution of 382 exonic nonsynonymous SNVs in six primary tumor regions, these mutations are mapped to 35 trunk mutations (54.97%, 210/382), 17 branch mutations (16.84%, 64/382), and 108 private mutations (28.27%, 108/382). The heat map reveals the presence (*red*) or absence (*blue*) of a mutation in each region of sample. The color bars above the heat map give a value (Var1) that indicates the number of samples involving the same mutation site

The gastric cancer patient was a 61-year-old male who underwent tumor-reductive surgery in July 2015. The postoperative pathological analysis showed that the tumor was a moderately differentiated adenocarcinoma with a bulge in the cardiac gastric gland. The tumor was 3 × 2.2 × 1.2 cm in size (Fig. 1a) and had invaded all layers of the stomach wall and lower esophagus. The lymph nodes exhibited no tumor cells metastasis.

Six multiregional samples were collected in a clockwise pattern from the surgically resected gastric carcinoma within 30 min after the surgery; the samples were immediately preserved in liquid nitrogen (Fig. 1b). The collection and use of the patient samples were approved by the Zhejiang Hospital of Traditional Chinese Medicine. Written informed consent was obtained from the participant for publication of this case report. We confirmed that all methods were performed in accordance with the relevant guidelines (Approved Guidelines of the Clinical and Laboratory Standards Institute MM01-A3, MM13-A, and MM20-A, and the CARE Guidelines).

## Methods

### Multiregional WES

DNA was extracted from six samples of one gastric carcinoma tumor. Exomes were captured from 750 ng of genomic DNA per sample using the Agilent SureSelect Human All Exon V5 Kit (Agilent Technologies, Santa Clara, CA, USA) according to the manufacturer’s instructions. Paired-end multiplex sequencing was then performed on the Illumina HiSeq X10 sequencing platform. On average, the sequencing depth was 161× per sample (ranging from 133× to 216× with a standard deviation of ±28×).

### Identification of somatic mutations

Somatic mutations were identified by the pipeline following the Genome Analysis Toolkit (GATK) best practices for somatic SNV and INDEL discovery in WES [24–26]. Our analysis was mainly focused on exonic mutations, including nonsynonymous SNVs, synonymous SNVs, small INDELs, and stop codon mutations (stopgains). Paired-end reads in the FastQ format were aligned to the reference human genome (GRCh37) using Burrows-Wheeler Aligner (BWA-MEM) with the default settings [27]. The aligned reads were further processed by sorting, duplicate removal, INDEL realignment, and base recalibration using Samtools [28], Picard-tools and GATK [24]. Somatic SNVs and small INDELs were detected using MuTect2 [25], which is a built-in package in GATK v3.5. In addition to MuTect2’s built-in filters, we applied the following filtering criteria for SNVs: (i) total read count in tumor DNA ≥ 50; (ii) total read count in germline DNA ≥ 30; (iii) VAF in tumor DNA ≥ 5%; (iv) VAF in blood DNA = 0. The following criteria for small INDELs: (i) total read count in tumor DNA ≥ 10; (ii) total read count in blood DNA ≥ 6; (iii) VAF in tumor ≥ 5% and total number of reads supporting a call ≥ 5; and (iv) VAF in normal DNA = 0. SNVs and INDELs were annotated using ANNOVAR [29]. Primers were designed to validate the 27 trunk nonsynonymous SNVs shared by the six samples using PCR and sequencing.

Moreover, another set of raw data from the somatic mutations of multiregional samples of 11 surgically resected lung adenocarcinomas [15] was downloaded and analyzed using the same pipeline to investigate the distribution of the mutations across samples. The VAFs of the nonsynonymous SNVs in gastric carcinoma were analyzed based on the relationships between the trunk, branch and private branch mutations, using the Wilcoxon test implemented in R (https://www.r-project.org/).

The UniProtKB/Swiss-Prot (http://www.uniprot.org) and DAVID v6.8 [30] were utilized for functional annotation analysis of mutated genes with trunk, branch and private branch mutations.

### Phylogenetic analysis

The somatic mutation profile for each tumor sample was converted into binary format. All somatic mutations that were present in the exonic regions of at least one tumor sample were used for the phylogenetic analysis. The germline DNA from the blood sample was set as the outgroup with the assumption of somatic mutations. The phylogenetic tree was inferred with the neighbor-joining method using MEGA7 [31].

## Results

### Identification of somatic mutations by multiregional WES

To evaluate the ITH in gastric carcinoma, multiregional WES was performed on tumor genomic DNA obtained from a patient with gastric carcinoma (Fig. 1a, b); normal genomic DNA was extracted from blood as a reference. WES was conducted at a mean depth of 161×. Across all six regions of the tumor, 1231 simple somatic mutations, including SNVs and small INDELs (Additional file 1), were identified, and these mutations included 539 exonic mutations consisting of nine INDELs, 20 stopgains, 128 synonymous SNVs and 382 nonsynonymous SNVs (Additional file 2: Table S1). The distribution of SNVs in the protein-coding regions, including stopgains as well as synonymous and nonsynonymous SNVs, indicates the phylogenetic relationship across the six samples (Fig. 1c). Most of the somatic mutations in protein-coding regions either occur in only one sample (28.76%, 155/539) or are common to all samples (53.42%, 288/539) (Fig. 1d, Additional file 3: Fig. S1 A-B). All 382 of the nonsynonymous SNVs from six samples originated from 160 exact mutation sites (Additional file 4), including 210 nonsynonymous SNVs derived from 35 common mutation sites in all six samples that were classified as trunk mutations (54.97%, 210/382). Non-trunk mutations did not occur in all of the samples, among these mutations, 108 nonsynonymous SNVs occurred in only one sample and were classified as private branch mutations (28.27%, 108/382). The other 64 nonsynonymous SNVs were branch mutations that were present in some but not all regions of the tumor.

The 35 trunk mutations occurred on 35 genes, and these genes were called trunk genes; similarly, the private and branch genes were defined as well. For example, SPEN, PRODH2, IGSF10, ITK and MTNR1B were five of the trunk genes, and SMARCE1 was a branch gene harboring mutations from two different sites that was involved in four and two samples. SPEN, PRODH2, IGSF10, MTNR1B and SMARCE1 were found to be mutated in 0.49% (1/203) of esophageal cancers, 0.41% (1/246) of endometrial cancers, 0.52% (1/194) of cervical cancers, 0.26% (1/391) of pancreatic cancers and 0.55% (1/183) of melanomas, in the International Cancer Genome Consortium (ICGC) database (http://icgc.org/, release 22). An additional eight genes were also found in the ICGC database (Additional file 2: Table S2). Notably, two trunk genes (SPEN and ITK), a branch gene (SMARCE1), and several private genes (FAT4, CACNA1D, ATR, RUNX1T1, TERT, and SRGAP3) were defined as cancer-associated genes, according to the cancer gene census [32, 33]. SPEN supports the transcription activation in osteoblasts and is an essential corepressor protein to regulate different key pathways, including the Notch pathway. SPEN can block the precursor B cells differentiating into marginal zone B cells and also repress the transcription via the recruitment of large complexes that contain histone deacetylase proteins [34]. ITK is a tyrosine kinase that plays an essential role in the regulation of the adaptive immune response. ITK also regulates the development, function and differentiation of conventional T cells and nonconventional NKT cells [35]. SMARCE1 is involved in the transcriptional activation and repression of select genes, and also in the repression of neuronal specific gene promoters in non-neuronal cells through specifically interaction with the CoREST corepressor [36]. Function of the remaining cancer-associated genes and all mutated genes is included in Additional file 2: Table S3 and Additional file 5.

Twenty-seven of 35 trunk mutations were verified by PCR amplification, and 24 trunk mutations were verified in all six tumor samples. However, one mutation in RBM12 was verified in only one sample, and two mutations in SPEN and TWIST2 failed to be verified due to unavailable rational primers. None of the mutations were verified in the blood samples (Additional file 3: Fig. S1C).

### Multiregional sequencing can identify the majority of trunk mutations

**Fig. 2:**
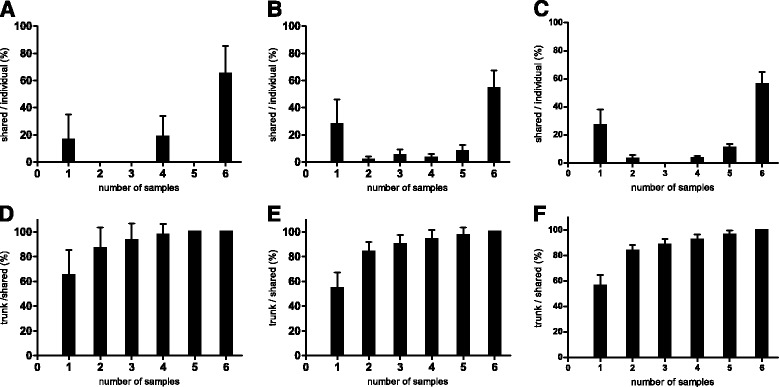
Common mutation proportions between regional samples and the number of samples sufficient to identify a majority of trunk mutations. **a** The proportion of stopgains shared by any one to six samples in the respective samples. **b** The proportion of synonymous mutations shared by any one to six samples in the respective samples. **c** The proportion of nonsynonymous mutations shared by any one to six samples in respective samples. The x-axis indicates the volume of samples needed to result in shared mutations. **d**-**f** The proportion of trunk mutations (*n* = 35) of the mutations shared by any one to six samples. The x-axis indicates the volume of samples needed to result in shared mutations. (**d**: stopgains, **e**: synonymous SNVs, **f**: nonsynonymous SNVs). Two-region sampling (83%) or three-region sampling (89%) may be a cost-effective strategy to obtain a majority of trunk mutations in gastric carcinoma

In this study, we focused on nonsynonymous SNVs for further analysis because nonsynonymous SNVs could generate tumor-specific mutant proteins. These mutations were shared among the samples (Fig. 2a-c). Almost all of the mutations either occurred in a single sample or were shared by all six samples, in agreement with the heat maps (Fig. 1c, Additional file 3: Fig. S1 A-B). For each sample, trunk mutations made up a large proportion, ranging from 42.68% to 70.00%, followed by private mutations (12.00%–45.12%). Private mutations show the degree of ITH, while trunk mutations indicate some homogeneity. We found that approximately 83% of the common mutations of any two samples and approximately 89% of the common mutations of any three samples were trunk mutations (Fig. 2d-f), which indicated that sampling of two or three regions could identify the majority of trunk mutations in the gastric carcinoma of the patient.

**Fig. 3:**
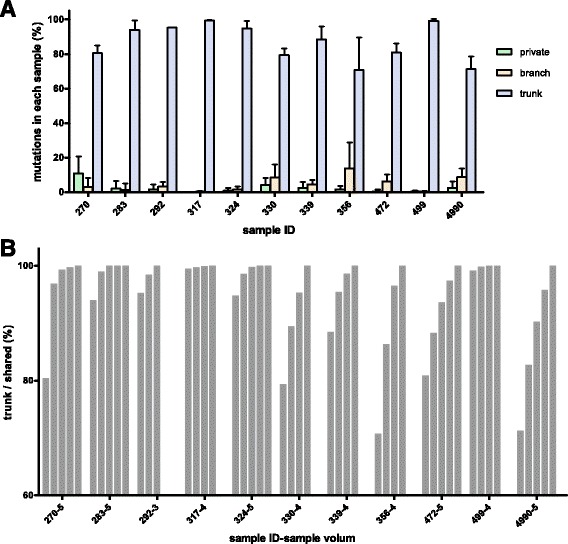
Mutation proportions of lung adenocarcinomas across samples. **a** Distribution of exonic nonsynonymous SNVs in 11 tumors. The color bars indicate classification of mutations according to whether they are trunk (*blue*), branch (*pink*) or private (*green*) mutations. The x-axis shows the sample IDs of the 11 tumors. **b** The proportion of trunk mutations among the shared mutations of any set of samples, from one to maximum volume from each tumor. The x-axis indicates sample IDs with sample volumes. Detailed information is shown in Additional file 3: Fig. S3

To explore the effectiveness of small-scale multiregional sampling in the identification of trunk mutations, we conducted a secondary analysis of a published dataset on a different cancer type, which was a multiregional WES of 48 regions from 11 resected lung adenocarcinomas [15]. The exonic nonsynonymous SNVs from the somatic mutations of 48 samples were analyzed in the same way as in the gastric carcinoma study. We found that most mutations across each tumor sample were trunk mutations (Additional file 3: Fig. S2). On average, 86.29% of the exonic nonsynonymous SNVs were trunk mutations (Fig. 3a). More than 82% of the shared mutations of any two samples and more than 90% of the shared mutations of any three samples were trunk mutations (Fig. 3b). This result is consistent with the results of the gastric carcinoma study.

### Trunk mutations tend to have higher variant allele frequencies

**Fig. 4:**
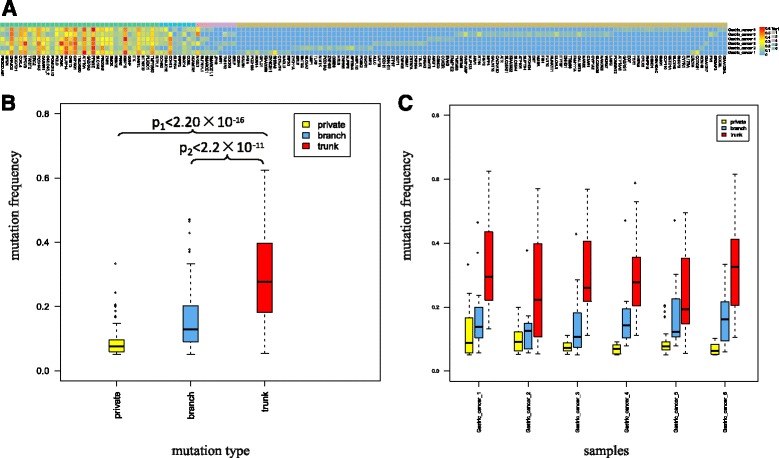
Mutation frequency distribution in multiregional samples of gastric carcinoma according to mutation types. **a** Distribution of exonic nonsynonymous mutations in each region of sample with mutation frequency. **b** Mutation frequencies of all exonic nonsynonymous mutations, classified as private (*yellow*), branch (*blue*), and trunk (*red*) mutations. p_1_ shows a significant difference between the trunk and private mutations, and p_2_ shows a significant difference between the trunk and branch mutations. **c** Mutation frequency of three types of mutations in each sample. Gastric cancer 1 (p_1_ = 3.5 × 10^−6^, p_2_ = 2.4 × 10^−4^), gastric cancer 2 (p_1_ = 1.6 × 10^−4^, p_2_ = 3.8 × 10^−2^), gastric cancer 3 (p_1_ = 6.5 × 10^−8^, p_2_ = 1.9 × 10^−5^), gastric cancer 4 (p_1_ = 5.4 × 10^−5^, p_2_ = 2.7 × 10^−3^), gastric cancer 5 (p_1_ = 5.2 × 10^−8^, p_2_ = 9.5 × 10^−2^), gastric cancer 6 (p_1_ = 8.6 × 10^−10^, p_2_ = 9.3 × 10^−4^). The *p* values were determined with a Wilcoxon test

We further analyzed the variant allele frequencies (VAFs) of the exonic nonsynonymous SNVs for the trunk and non-trunk mutations in gastric carcinoma. Most of the high-VAF mutations were located at the trunk mutation area (Fig. 4a). Generally, the VAFs of the trunk mutations (mean value = 0.30) were significantly higher than those of the branch mutations (mean value = 0.17, *p* < 2.2 × 10^−11^) and private mutations (mean value = 0.09, *p* < 2.2 × 10^−16^) (Fig. 4b). The VAFs in each sample also suggested that trunk mutations (mean value ranged from 0.23 to 0.33) occurred much more frequently than branch and private mutations (mean value ranged from 0.14 to 0.18 and 0.07 to 0.12, respectively; Fig. 4c). However, the VAF of a certain mutation may vary in the same mutation type: the VAFs of trunk mutations varied from 0.12 to 0.52, and those of branch and private mutations varied from 0.05 to 0.47 and 0.05 to 0.33, respectively (Additional file 4). For example, the VAFs of trunk mutations in POM121L12 (mean VAF = 0.12) and RBM12 (mean VAF = 0.12) were less than the VAFs of private mutations in SHISA9 (VAF = 0.33) and CCDC91 (VAF = 0.24); this variation accounts for the observation that mutations with a high VAF are not always trunk mutations. Similarly, mutations with low VAF were not necessarily private mutations. Therefore, to identify trunk mutations, it is of great significance to utilize multiregional sequencing when considering mutations with high VAF as trunk mutations.

## Discussion and conclusions

It is widely accepted that cancer is a process of microevolution [37, 38]. During the evolution of cancer, cells experience a reiterative process of clonal expansion, diversification and selection [39]. Evidence of marked ITH shows the genetic diversity within cancer cells [15, 16, 19, 20] and suggests challenges to cancer precision medicine based on the mutational landscape portrayed by single-tumor biopsies [40]. Circulating tumor DNA (ctDNA) potentially reflects the biology of a cancer, which makes it a promising new biomarker for cancer diagnosis and prognosis [41, 42]. Searching for trunk mutations will contribute to expose potential targets in ctDNA for liquid biopsy. Moreover, with the development of targeted therapy and immunotherapy for cancer, tumor-specific mutated proteins, especially neoantigens, have attracted much attention due to their potential to be ideal targets for tumor immunotherapy [8, 10]. It is clinically important to determine whether the tumor-specific mutated proteins are present in all cancer cells; their presence may determine the outcome of target therapy for cancer. Therefore, searching for trunk mutations, which occur in tumor-initiating cells and are present in all cancer cells, is of critical importance for cancer genomics.

In this proof-of-concept study, we attempted to investigate the genetic heterogeneity and homogeneity of gastric carcinoma from one patient through multiregional WES. We are fully aware of the potential limitations imposed by the small sample size. Our preliminary results suggested that both genetic heterogeneity and homogeneity for missense mutations were present in multiregional samples of the gastric carcinoma. Each sample had different mutational landscape, while trunk mutations accounted for nearly half of all nonsynonymous somatic mutations for each sample. In any two samples, the proportion of trunk mutations among the shared mutations was approximately 83% on average. Approximately 89% of shared mutations were trunk mutations in any three samples. Although multiregional sampling, or more ideally single-cell sequencing, is believed to be needed to fully assess the complexity of ITH for each tumor, the number of samples required for sequencing is important for the practical consideration of balancing cost and sufficiency. This study suggests that small-scale multiregional sampling and subsequent screening of low-VAF somatic mutations might be a cost-effective strategy for identifying trunk mutations for gastric carcinoma and lung adenocarcinoma, based on analyses of one gastric carcinoma and published data from 11 lung adenocarcinomas. However, ITH patterns might differ between cancer types. Studies involving more cancer types and larger cohorts will lead to a more complete understanding of the biological and therapeutic impacts of ITH.

## Additional files

Additional file 1: List of simple somatic mutations across all six regions of the tumor, including SNVs and small INDELs. (XLSX 123 kb)

Additional file 2: Supplementary Table S1, S2, S3. (DOCX 23.5 kb)

Additional file 3: Supplementary Fig. S1-S3. (PDF 2620 kb)

Additional file 4: List of all 382 nonsynonymous SNVs from six samples originated from 160 exact mutation sites. (XLSX 21.7 kb)

Additional file 5: Function annotation tables of 160 mutated genes, including Interpro, Gene Ontology (GO), KEGG pathway, Online Mendelian Inheritance in Man (OMIM) according to the DAVID Bioinformatics Resources. (XLSX 57 kb)

## Abbreviations

BWA: Burrows-Wheeler Aligner
GATK: Genome Analysis Toolkit
ICGC: International Cancer Genome Consortium
INDELs: Insertions/deletions
ITH: Intratumor heterogeneity
SNVs: Single nucleotide variants
VAFs: Variant allele frequencies
WES: Whole-exome sequencing
