# Supplementary material for: Identification of trunk mutations in gastric carcinoma: a case study
Source: BMC Med Genomics. 2017 Jul 17;10:49. doi: 10.1186/s12920-017-0285-y (PMC5520061; doi:10.1186/s12920-017-0285-y)
Supplement: Supplementary file 2 — Supplementary Table S1, S2, S3. (DOCX 23.5 kb) [file 12920_2017_285_MOESM2_ESM.docx]

**Table S1.** **The number of somatic mutations in six samples**

| **Samples** | **#Somatic mutations** | **Exonic mutations** | | | |
| --- | --- | --- | --- | --- | --- |
|  |  | **#nonsynonymous SNVs** | **#synonymous SNVs** | **#stopgains** | **#INDELs** |
| Gastric cancer 1 | 267 | 62 | 31 | 3 | 1 |
| Gastric cancer 2 | 202 | 61 | 18 | 2 | 0 |
| Gastric cancer 3 | 177 | 63 | 21 | 3 | 2 |
| Gastric cancer 4 | 155 | 50 | 16 | 3 | 3 |
| Gastric cancer 5 | 224 | 82 | 25 | 5 | 0 |
| Gastric cancer 6 | 206 | 64 | 17 | 4 | 3 |
| Total | 1231 | 382 | 128 | 20 | 9 |

**Table S2.** **Genes involved in 160 mutation sites recorded in ICGC (release 22)**

| **Gene** | **Cancer type** | **ICGC ID** | **Consequence** | **Donors affected** | **Across all projects** |
| --- | --- | --- | --- | --- | --- |
| SPEN | Esophageal cancer | MU34462011 | R810W,2428C>T | 1 / 203 (0.49%) | 1/10638(0.01%) |
| PRODH2 | Endometrial cancer | MU1922768 | A367V,1100C>T; A31V,92C>T | 1 / 246 (0.41%) | 1/10638(0.01%) |
| IGSF10 | Cervical cancer | MU28976482 | R729C,2185C>T | 1 / 194 (0.52%) | 1/10638(0.01%) |
| MTNR1B | Pancreatic cancer | MU7393190 | A135T,403G>A | 1 / 391 (0.26%) | 1/10638(0.01%) |
| SMARCE1 | Melanoma | MU42110151 | S2L,5C>T | 1 / 183 (0.55%) | 1/10638(0.01%) |
| DYNC2H1 | Endometrial cancer | MU1882498 | R3649H,10946G>A; R3656H,10967G>A | 1 / 246 (0.41%) | 1/10638(0.01%) |
| SNTG2 | Colon cancer | MU66240 | A448T,1342G>A; A321T,961G>A | 1 / 216 (0.46%) | 1/10638(0.01%) |
| SLC5A7 | Colorectal cancer  Cutaneous melanoma | MU4465083 | R382Q,1145G>A; R277Q,830G>A | 1 / 187 (0.53%)  2 / 335 (0.60%) | 3/10638(0.03%) |
| LRP1B | Esphageal cancer  Gastric cancer | MU5591283 | Q156H, 468A>C | 1 / 203 (0.49%)  1 / 289 (0.35%) | 2/10638(0.02%) |
| MPP4 | Colorectal cancer  Colon cancer  Endometrial cancer | MU149934 | E470K,1408G>A; E463K,1387G>A; E446K,1336G>A; E412K,1234G>A; E426K,1276G>A; E395K,1183G>A | 1 / 187 (0.53%)  1 / 216 (0.46%)  1 / 246 (0.41%) | 3/10638(0.03%) |
| POMGNT2 | Renal cancer | MU3899084 | R141H. 422G>A | 1 / 10 (10.00%) | 1/10638(0.01%) |
| CSMD1 | Endometrial cancer  Esophageal cancer | MU1859714 | V2150I,6448G>A; V2151I,6451G>A; V1630I,4888G>A | 2 / 246 (0.81%)  1 / 228 (0.44%) | 3/10638(0.03%) |
| FN1 | Breast cancer | MU66014065 | R2018C,6052C>T; R1928C,5782C>T; R46C,136C>T; R1927C,5779C>T; R644C,1930C>T; R1837C,5509C>T | 2/ 624 (0.32%) | 2/10638(0.02%) |

**Table S3. Gene functions of mutated genes reported as cancer-associated genes in the cancer gene census**

| **Classification** | **Gene** | **Gene function** |
| --- | --- | --- |
| Trunk mutation | SPEN | SPEN supports the transcription activation in osteoblasts and is an essential corepressor protein to regulate different key pathways, including the Notch pathway. It could block the precursor B-cells differentiating into marginal zone B-cells, and also repress the transcription via the recruitment of large complexes that contain histone deacetylase proteins. (PMID: 12374742) |
|  | ITK | ITK is a tyrosine kinase and plays an essential role in regulation of the adaptive immune response. ITK also regulates the development, function and differentiation of conventional T-cells and nonconventional NKT-cells. (PMID: 12682224) |
| Branch mutation | SMARCE1 | It’s involved in the transcriptional activation and repression of select genes, and also in the repression of neuronal specific gene promoters in non-neuronal cells through specifically interaction with the CoREST corepressor. (PMID: 23377182) |
| Private mutation | FAT4 | FAT4 plays a role in the maintenance of planar cell polarity as well as in inhibition of YAP1-mediated neuroprogenitor cell proliferation and differentiation. (PMID: 15003449) |
|  | CACNA1D | CACNA1D mediates the entry of calcium ions into excitable cells and is also involved in a variety of calcium-dependent processes, including muscle contraction, hormone or neurotransmitter release, gene expression, cell motility, cell division and cell death. (PMID: 18482979) |
|  | ATR | Serine/threonine protein kinase, which activates checkpoint signaling upon genotoxic stresses such as ionizing radiation (IR), ultraviolet light (UV), or DNA replication stalling, thereby acting as a DNA damage sensor. ATR mutations ATR mutations play an important role in the development and clinical behavior of a subset of microsatellite instability–positive endometrial, colon, and stomach cancers (PMID:16103057, 11691784) |
|  | RUNX1T1 | Transcriptional corepressor, it facilitates transcriptional repression via its association with DNA-binding transcription factors and recruitment of other corepressors and histone-modifying enzymes. (PubMed:12559562, 15203199). |
|  | TERT | Telomerase reverse transcriptase, a ribonucleoprotein enzyme essential for the replication of chromosome termini in most eukaryotes. Active in progenitor and cancer cells. Inactive, or very low activity, in normal somatic cells. (PMID: 14963003) |
|  | SRGAP3 | SLIT-ROBO Rho GTPase-activating protein 3, a GTPase-activating protein for RAC1 and perhaps Cdc42, but not for RhoA small GTPase. May attenuate RAC1 signaling in neurons. (PMID: 12195014) |
