# Supplementary material for: Identification of trunk mutations in gastric carcinoma: a case study
Source: BMC Med Genomics. 2017 Jul 17;10:49. doi: 10.1186/s12920-017-0285-y (PMC5520061; doi:10.1186/s12920-017-0285-y)
Supplement: Supplementary file 3 — Supplementary Fig. S1-S3. (PDF 2620 kb) [file 12920_2017_285_MOESM3_ESM.pdf]

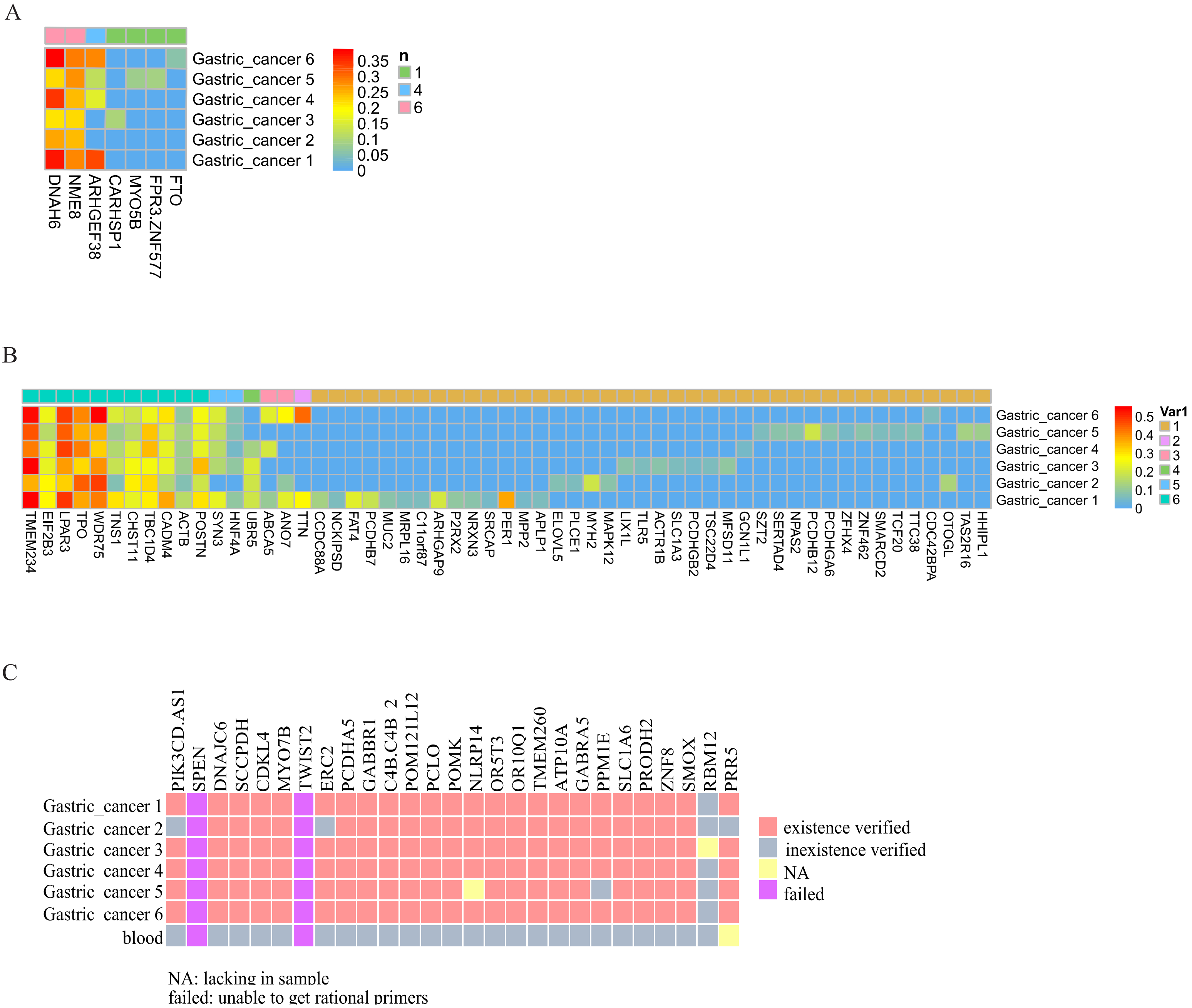

Figure S1. Mutation frequency of stopgains and synonymous SNVs across samples, and verification of 27 trunk mutations.
(A) Mutation frequency of stopgains. (B) Mutation frequency of synonymous SNVs. The colorramp shows the range of mutation frequency. n&Var1: the number of samples involved in each mutation. (C) Verification of 27 nonsynonymous trunk mutations by PCR amplification and sequencing.

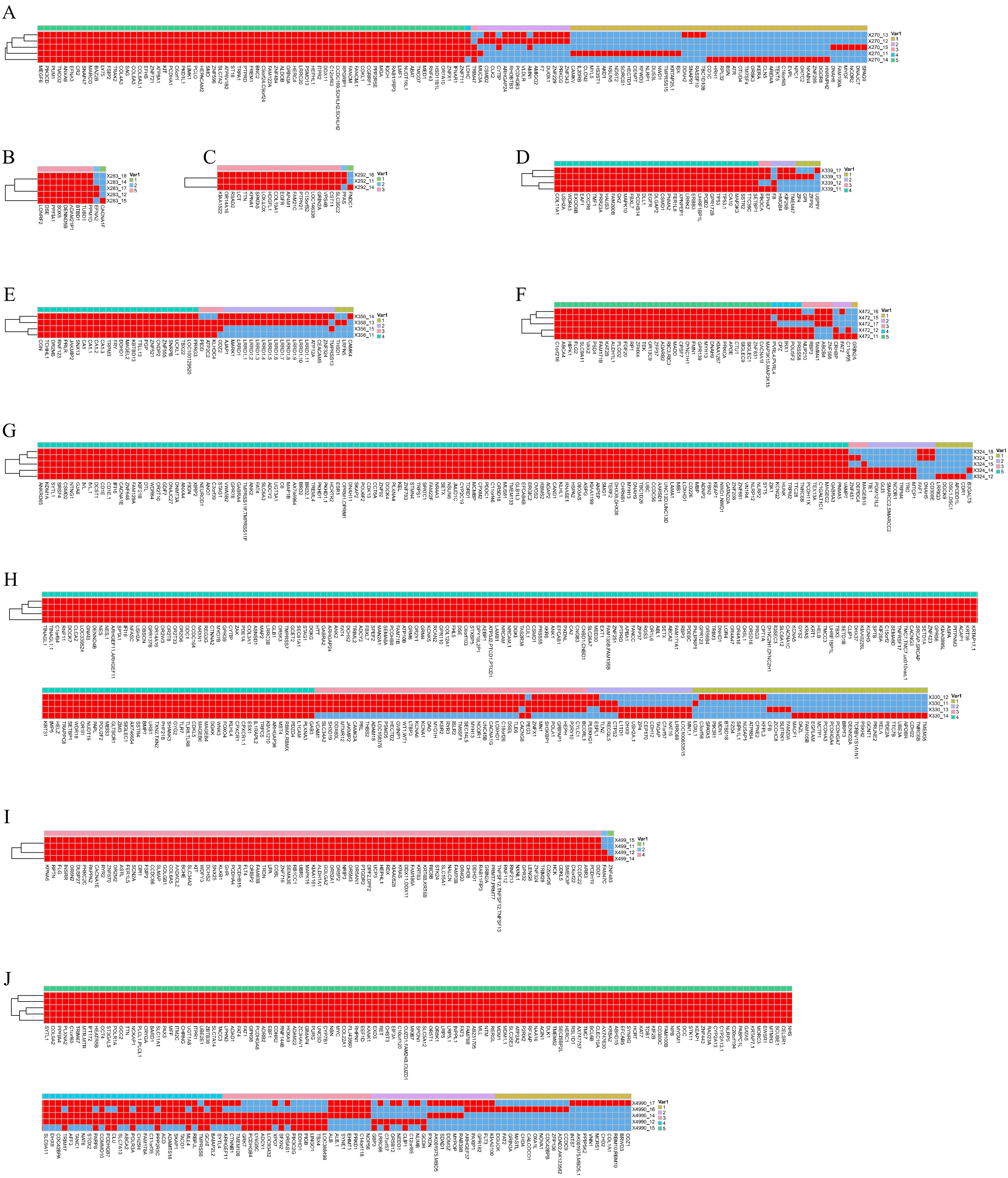
A
B
C
D
E
F
G
H
I
J

K

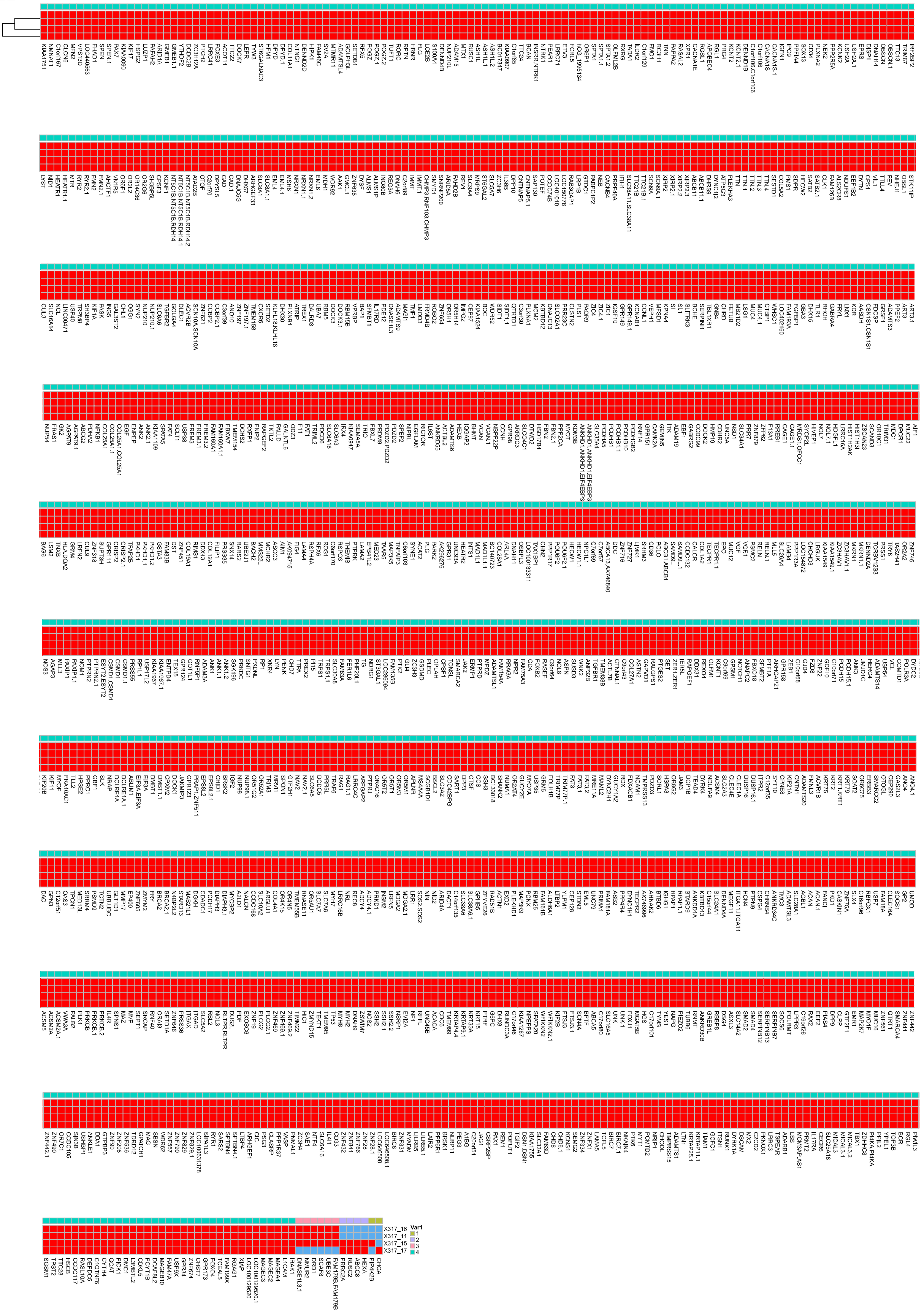

Figure S2. Distribution of exonic nonsynonymous SNVs of 11 lung adenocarcinomas across samples.

A-K: Distribution of mutations across samples from 11 lung adenocarcinomas, x(n)_(n): sample id_region id, var1: the number of samples involved in each mutation. Samples were clustered in each tumor.

A B

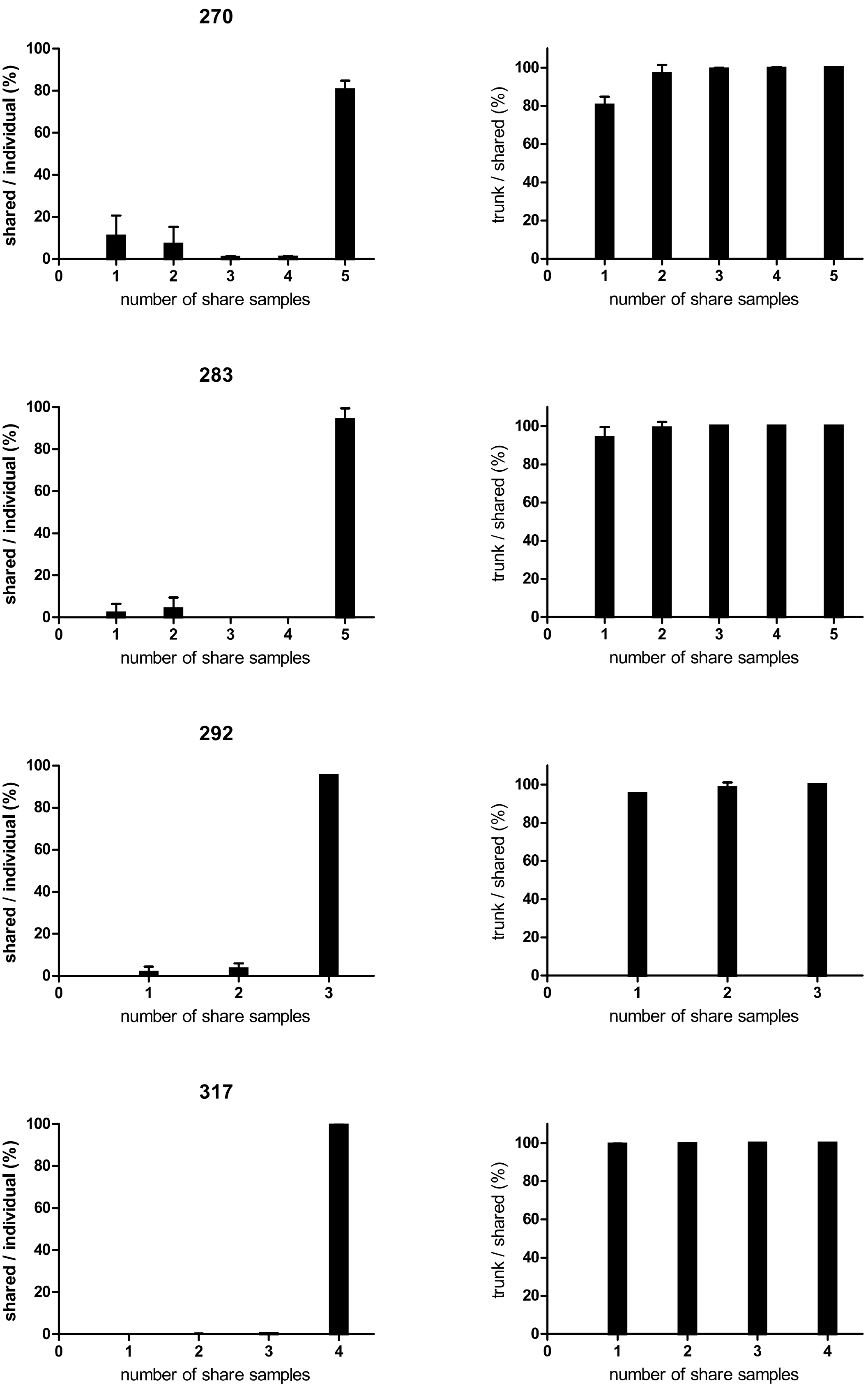

270
283
292
317
shared / individual (%)
trunk / shared (%)
number of share samples

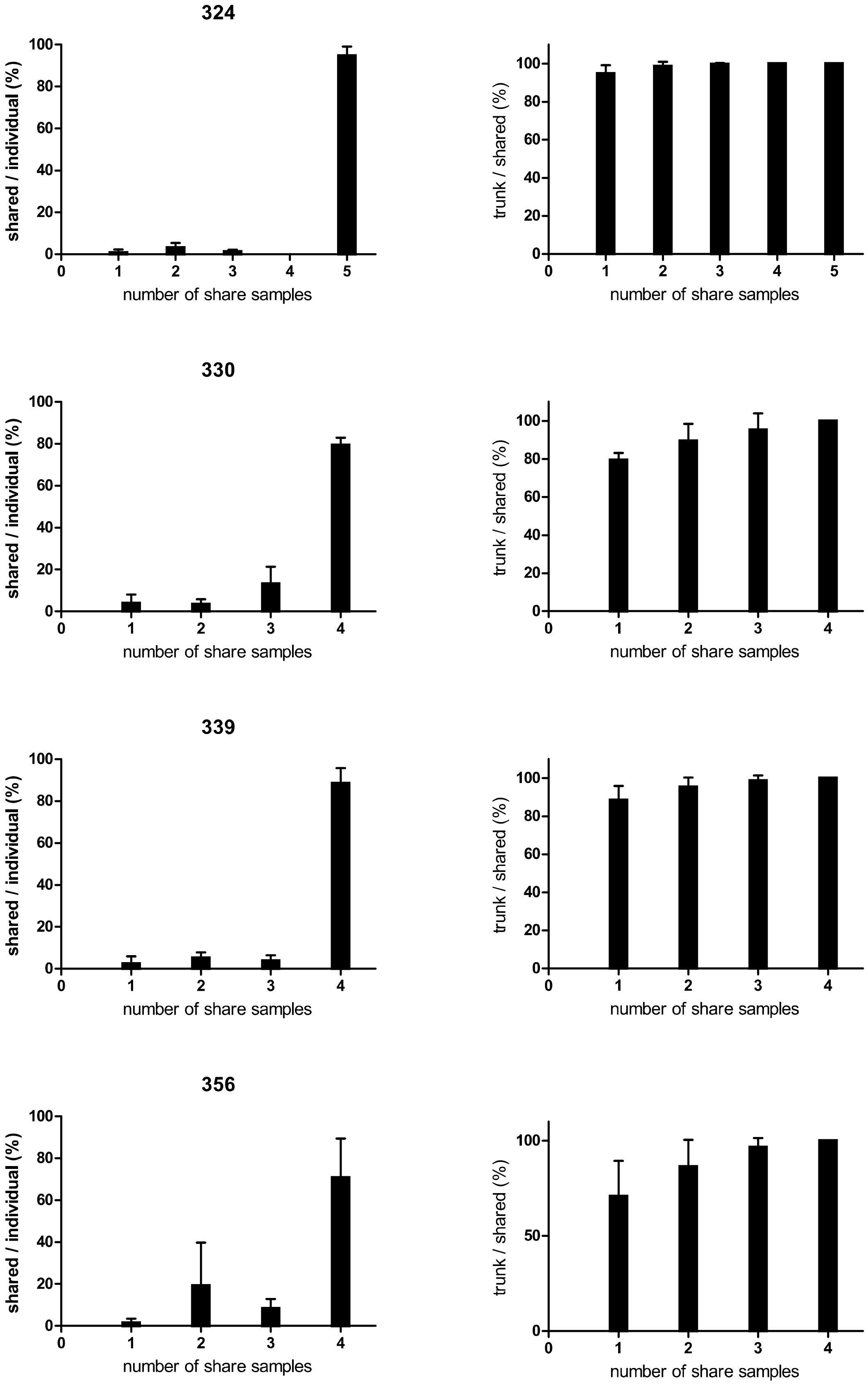

324
330
339
356
shared / individual (%)
trunk / shared (%)
number of share samples

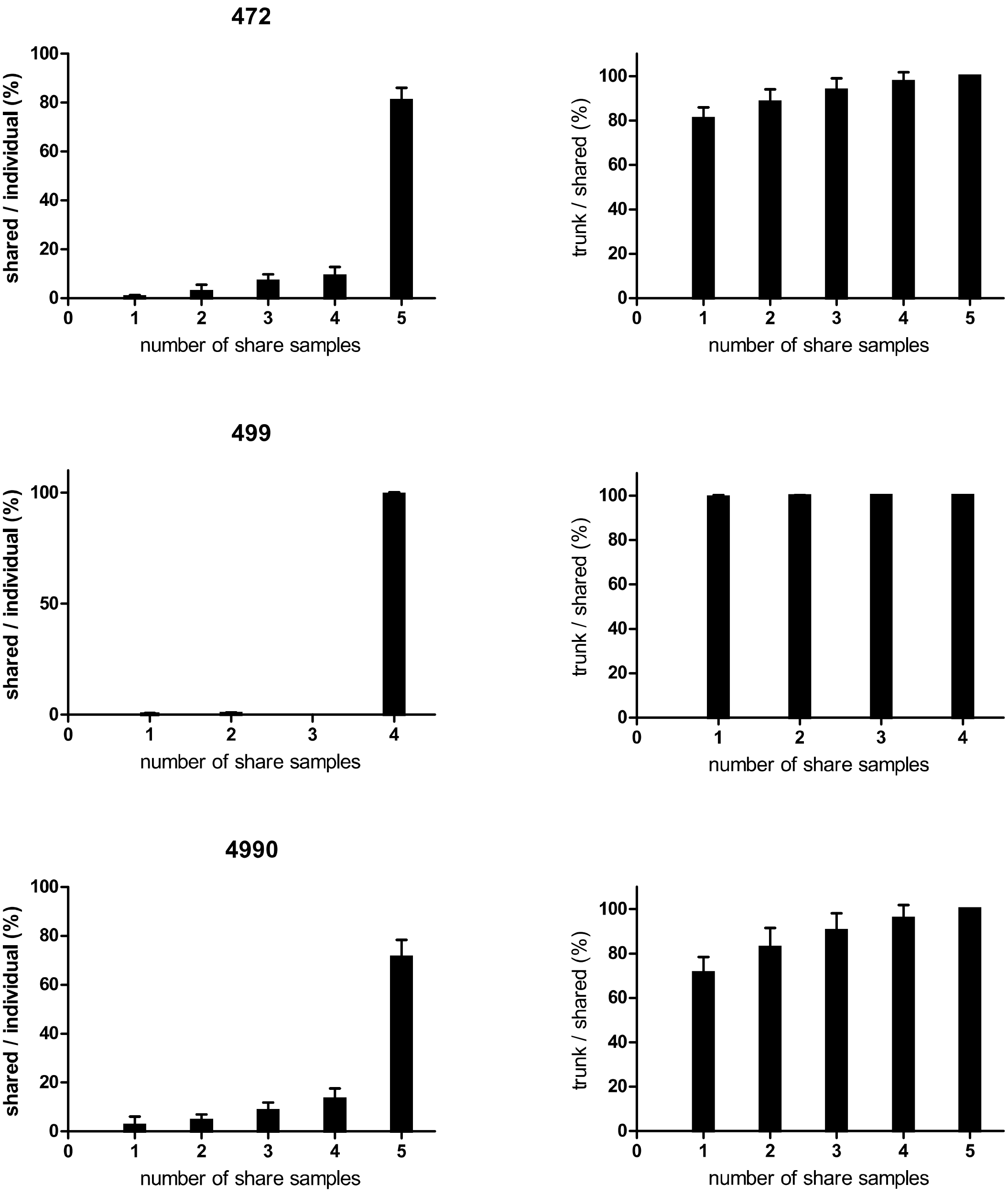

Figure S3. Mutation proportions of lung adenocarcinomas across samples.

Panel A shows the proportion of shared nonsynonymous mutations of any one to maximum volum of samples in respective samples, panel B shows the proportion of trunk mutations in shared mutations of any one to maximum volum of samples from each tumor. The x-axis indicate the volum of samples to give shared mutations.
